# Personality traits across countries: Support for similarities rather than differences

**DOI:** 10.1371/journal.pone.0179646

**Published:** 2017-06-16

**Authors:** Petri Kajonius, Erik Mac Giolla

**Affiliations:** 1 Department of Psychology, University of Gothenburg, Gothenburg, Sweden; 2 Department of Behavioral Sciences, University West, Trollhättan, Sweden; 3 Department of Cognitive Neuroscience and Philosophy, University of Skövde, Skövde, Sweden; Universitat Wien, AUSTRIA

## Abstract

In the current climate of migration and globalization, personality characteristics of individuals from different countries have received a growing interest. Previous research has established reliable differences in personality traits across countries. The present study extends this research by examining 30 personality traits in 22 countries, based on an online survey in English with large national samples (*N*_Total_ = 130,602). The instrument used was a comprehensive, open-source measure of the Five Factor Model (FFM) (IPIP-NEO-120). We postulated that differences in personality traits between countries would be small, labeling this a Similarities Hypothesis. We found support for this in three stages. First, similarities across countries were observed for model fits for each of the five personality trait structures. Second, within-country sex differences for the five personality traits showed similar patterns across countries. Finally, the overall the contribution to personality traits from countries was less than 2%. In other words, the relationship between a country and an individual’s personality traits, however interesting, are small. We conclude that the most parsimonious explanation for the current and past findings is a cross-country personality Similarities Hypothesis.

## Introduction

With migration and globalization, individual differences across countries have become a topic of interest and importance. Large-scale studies propose that country differences in personality traits are significant, and related to societal values [1]. This could potentially provide insights into the mindset of both individuals and cultures. However, another way of interpreting past findings is that aggregate trait-personality differences between countries are notably small. Building on this train of thought, we propose and test a Similarities Hypothesis, defined as personality traits being similar in structure and sex differences across countries, as well as in aggregate country levels. In the present study, we ask (i) to what degree does personality data fit the five trait factor-structures across countries? (ii) to what extent do personality traits predict sex differences across countries? and (iii) how much do personality trait levels differ across countries? For this purpose, we make use of a comprehensive, open-source personality instrument, based on the well-known Five Factor Model (FFM) [2], which includes the measurement of 30 personality trait facets: IPIP-NEO-120 [3]. This was tested on some of the largest country samples to date (*N* > 1,000), from 22 countries world-wide (*N*_Total_ = 130,602). There are at least half a dozen large-scale cross-country studies to date. However, these often used modest country sample sizes, sometimes with equivocal results (see a comprehensive review [4]).Generally speaking, it is still a largely unsettled issue how reliable, valid and consequential country differences in personality traits are. In the wake of the suggested replication crisis within psychology [5], it is continually necessary to reaffirm psychological foundations such as cross-country personality measurement.

### Geographical differences in personality traits

Personality traits refer to recurring ways of thinking, feeling, and behaving, which show heritability and stability across time [6]. A growing consensus accepts the Five Factor Model (FFM) as a reasonable conceptualization of human personality. The FFM consists of the five trait factors: Neuroticism (N); Extraversion (E); Openness (O); Agreeableness (A); and Conscientiousness (C). In one of the original and most used FFM instruments, NEO-PI-R [7], the five trait factors are composed of 30 specific trait facets. Extraversion, for instance, is composed of facets such as friendliness, cheerfulness, and assertiveness, while Conscientiousness is composed of facets such as self-efficacy, orderliness and dutifulness. These specific traits are in turn informed by four separate questionnaire items, which also have shown trait-like features such as heritability and stability [8]. For a full list of the 30 facet-traits investigated in the present study, see Table 3.

Research suggests that personality traits vary geographically [9]. For instance, neighborhoods in the greater London metropolitan area have been shown to be separated by FFM levels—more open-minded, extraverted, and less agreeable people live closer to the city center. Furthermore, these geographical trait differences demonstrate predictive validity on life outcomes, such as happiness and socio-economic affluence [9]. Trait differences extend into the regions of Great Britain, with higher agreeableness in rural areas compared to large cities [9]. Corresponding findings have been found with samples from the US [10]. For instance, state level differences show the West coast to be characterized by high Openness and low Neuroticism, the Mid-West by high Agreeableness, and the East coast by comparatively higher Neuroticism and lower Conscientiousness [11].

### Cross-cultural equivalence

A growing body of evidence speaks for the usefulness of the Five Factor Model in measuring personality across countries and ethnic groups, suggesting that personality to a degree is cross-culturally equivalent [12]. For instance, samples from countries as diverse as Japan, Germany, and Canada, exhibit a near universal five factor structure based on genotype [13]. Further support comes from studies demonstrating that sex differences in personality traits have been found to replicate well across countries. For instance, self-report measures have established higher Neuroticism and agreeableness in women across 56 cultures [14]. These findings are supported by cross-cultural studies using observational methods [15].

However, the universality of the FFM has also been questioned. Researchers, for example, were unable to replicate the five factor structure in illiterate, indigenous farmers in the Amazon [16]. Only two out of five traits, *pro-sociality* (cf. Agreeableness) and *industriousness* (cf. Conscientiousness) could be established. In other studies, only three factors, *affiliation* (cf. Agreeableness), *order* (cf. Conscientiousness), and *dynamism* (cf. Extraversion) have been replicated [17, 18, 19]. Such contrary findings continue to drive the scrutiny and testing of personality measurement [20].

Even if a universal FFM structure can be assumed, criticism may rightly be raised to what extent the FFM measurement works similarly across countries. For an instrument to be measurement invariant, and thus enable meaningful comparisons across countries, statistical constructs as well as intercepts in various country populations need to be equivalent. Put simply, instruments need to measure the same constructs (*construct bias*) on the same scale (*method bias*), across groups. Even though a majority of large-scale studies conclude that there is a sufficient cultural equivalence in the FFM [21], measurement invariance in the strictest sense is seldom found. For example, in a comparison between US and Dutch respondents, up to 1/5 of items in respective FFM traits showed some differential item functioning (DIF) [22]. Relatedly, another study was unable to establish measurement invariance using a 36-item instrument across samples in 26 countries [18]. Nevertheless, the working assumption is that the FFM structure is *sufficiently* equivalent to enable comparisons of personality traits between countries [4].

### Cross-country studies

One of the largest cross-country personality surveys to date was conducted in 31 countries with a total sample size of over 1 million [1]. This study made use of a type of FFM with the 32-item Occupational Personality Questionnaire (OPQ32), and provided support for between-country differences in personality relating to cultural values. One of the early landmark, large-scale studies using the original 240-item NEO-PI-R version of the FFM mapped out the basic temperament traits, Neuroticism and Extraversion across 36 cultures (*N* = 27,965) [23]. One of its merits was the use of a variety of cultures within countries, such as Uralic, Sino-Tibetan, and Bantu, while one of its downsides was the use of primarily homogenous college samples. East Asian countries were lower in Extraversion and higher in Neuroticism compared to northern European countries. Another well-cited study made use of specific trait-facets in the NEO-PI-R and utilized observer-reports [21]. The observer reports, particularly for Extroversion, showed high correlations (*r* = .68) with self-reports across 26 nations. Country differences in personality have also been reported using brief FFM instruments, such as the BFI-44 [24]. This study was based on samples from 56 countries, covering 5 continents and 28 languages. However, country samples were often small (*N* < 100) and scale reliabilities at times low (α < .60). One conclusion based on these and other studies is that personality on regional or country levels can predict relevant country outcomes, such as societal values or economic output [1].

However, studies establishing personality differences across countries have been met with criticism. In a review of the extant research, Meisenberg [4] concluded that personality differences between countries are generally small and carry limited reliability and validity. Taken together, we may not yet know to what degree variations in personality reflect meaningful between-country differences rather than study artefacts.

### The current study

The present study represents some of the largest national samples to date (*N* > 1,000) using an extensive measure of the FFM (IPIP-NEO-120) [5], from 22 countries. First, we report model fit indices for the five trait factor structures for each country. This, to our knowledge, has only sparsely been reported in previous research, as model fits for personality structures has been a contentious issue [25]. Second, we report sex differences in personality traits for each country. Similar model fit indices and similar sex differences would imply that respective trait factor functions similarly across countries. Third, we report and compare mean levels of personality traits across countries. The overall hypothesis was that personality traits would show similar patterns across countries in terms of model fit and sex differences, and would vary little in terms of aggregate country levels. We name this a Similarities Hypothesis. Specifically, based on recommendations from standardized effect-sizes in meta-analyses [26], we defined similarity as less than 5% variance explained by country.

## Method

### Sample and procedure

A world-wide sample (*N* = 130,602) was selected from an extensive online personality survey given in English, collected by Prof. John Johnson [3]. The original data consisted of *N* = 619,150 representing most known countries in the world. Data was accumulated via a university web-site dedicated to research on personality. The site could be found via search engines and word-of-mouth, and attracted volunteers with the promise of brief but instant feedback based on the Five Factor Model. Respondents spent on average 20–30 minutes on the site. Every participant was informed that the questionnaire would be time-consuming, used for research purposes, and that careless responding would invalidate the usefulness of the data. No personal or traceable data, or data on socio-economic status, such as education or occupation, was collected or saved. The dataset is openly available to readers at https://osf.io/tbmh5/. The study followed the guidelines from the National Committee for Ethics and the Psychological Department at the University of Gothenburg, and the methods were approved at the time of data collection by the Pennsylvania State University. The data consisted solely of anonymous questionnaire data by active volunteers.

From the original data set of 619,150, we selected 19–69 year old respondents in countries which had sample sizes of *N* > 1,000, a limit where factor loadings start to stabilize [27]. This resulted in a total of 22 countries; representing many parts of the world (see Tables 1 and 2 for a full list). The sample consisted of 43% male (*N* = 55,334) and 57% female (*N* = 75,268) respondents, with an average age of 28.0 years (*SD* = 9.2). The descriptive sex data and standardized mean five factor trait levels are summarized in Table 2. (S1 Table also holds the raw scores of means and standard deviations, as well as internal consistencies for all 30 facet-traits from all countries).

### Measurements

The personality questionnaire used was the IPIP-NEO-120. This is an open-source adaptation of the widely used original NEO-PI-R [7, 28]. The IPIP-NEO measures 30 facet-traits with 120 items [3] and is based on the publically available international personality item pool (IPIP) [28]. IPIP-NEO-120 has demonstrated an overall ICC correlation profile with NEO-PI-R of *r* = .98 [29]. Each facet-trait is composed of 4 items measured on a 1–5 scale, and each of the five trait factors is in turn composed of six facet-traits. The mean Cronbach’s alpha reliability for the five trait factors was high (N = 0.90, E = 0.89, O = 0.81, A = 0.85, C = 0.90). Country belonging was formulated as, “Please indicate the country to which you feel you belong the most, whether by virtue of citizenship, length of residence, or acculturation.”

### Statistical analyses

Duplicates (people taking the test twice) and participants with repetitive patterns longer than 7 items were removed (<1% of the total sample). Missing data (<1% of the total sample) was corrected by imputing item means. Due to the large sample sizes, statistical analyses with and without data cleaning showed virtually identical results.

We used confirmatory factor analysis (CFA), in a structural equation model (SEM) framework, to examine trait-structures. An alternative option would have been to use a more exploratory framework, such as reporting congruence coefficients. However, as this has been extensively reported in previous research [30], it was deemed that model fit indices would be a greater contribution. The root mean square error approximation (RMSEA) was the primary fit index. This measure has the advantage of being more insensitive to large sample sizes (compared to for instance χ^2^). According to guidelines, RMSEA scores should preferably not be above .08 [31]. We also used the Comparative Fit Index (CFI) as a complementary statistic. A CFI above .90 or .95 is said to indicate adequate fit [31]. However, cut-off points should always be interpreted carefully since measurements and samples can differ considerably, and it is particularly problematic to achieve good CFI fits in hierarchical personality models [25]. For the sake of parsimony, we report unconstrained models that do not make use of covariance error residuals or modification indices. As there are reasons to believe that there are trait-levels below facets on item-level [8], an alternative would have been to correlate residuals, but it was decided that this would complicate the parsimony and current use of the FFM.

We used standardized mean differences (Cohen’s *d*) to examine sex differences in personality traits within countries. Based on meta-analytic recommendations [26], a *d* = 0.50 was considered to be an impacting effect. This effect size can be translated into a Pearson’s correlation of near *r* = .25.

To quantifying personality trait levels across countries, we compared and illustrated standardized z-scores of the 30 trait-facets for each country. Furthermore, we quantified the between-country (*N* = 22) and the within-country (*N* = 130,602) variance with help of ANOVA-models, one for each of the 30 facet-traits. We controlled for sex as a covariate. Interaction effects were not included due to lack of theoretical grounds, as well as the consistent near null-effects (age was excluded for similar reasons). Explained variances in facet-trait levels due to country belonging and sex are reported with eta-squared values. 5% explained variance was chosen as a cut off for a meaningful difference, based on recent recommendations [26]. In other words, explained variance below .05 would provide support for a Similarities Hypothesis.

## Results

Overall, as reported in S1 Table, among the five traits, Neuroticism showed the lowest mean scores and the highest variation, while Agreeableness the highest mean scores and the lowest variation across 22 countries. Cronbach’s alpha scores, which is one way to quantify scale reliability, showed similarities across countries: Openness showed the lowest reliabilities (average 0.81), ranging from 0.72 to 0.85, while Conscientiousness the highest (average 0.90), ranging from 0.86 to 0.92.

As an additional reliability-analysis, we compared our findings with the one previous large scale study using an equivalent NEO-based FFM instrument, [21]. The five factors (without correcting for error) exhibited correlations across 16 countries included in both studies: Neuroticism, *r* = .34, *p* < .05; Extraversion, *r* = .38, *p* < .05; Openness, *r* = .54, *p* < .01; Agreeableness, *r* = .32, ns; Conscientiousness, *r* = .39, *p* < .05.

First, five separate CFA models were conducted—one for each of the 5 hierarchical FFM trait structures. The trait structure is constituted by six specific facets, which in turn are constituted by 24 measurement items. Separate analyses, using Maximum Likelihood as the extraction method, were run for each country, resulting in 5 x 22 models in total, as reported in Table 1. All five trait-structures, with the exception of Neuroticism, overall converged without troubles. The structure of Neuroticism occasionally resulted in an initial Heywood case, which could be amended by constraining the very high loading from the facet-trait Anxiety. Table 1 summarizes the model fit indices, showing that the items and facet-traits overall converge into one factor across countries. Out of the 5 x 22 RMSEA scores, approximately 98% were found between the more liberal cut-off point of 0.08 for sufficient fit and the more conservative cut-off of 0.05 of excellent fit, with the remaining RMSEA scores being below 0.05. Hence, RMSEA scores suggest acceptable and similar fits across countries, in support of a Similarities Hypothesis. However, CFA fit indices were more meager. Particularly poor fits were observed for several East Asian countries. None of the CFI scores for Extraversion and Openness in the 22 countries were above the more liberal cut-off value of 0.90. For Agreeableness, only one country showed a CFI score greater than 0.90. Better fits, however, were observed for Neuroticism and Conscientiousness, with a large majority of countries showing CFI scores between 0.90–0.95.

**Table 1:** Model fits for the five personality trait factor-structures across countries.

Country	N		E		O		A		C	
	RMSEA	CFI	RMSEA	CFI	RMSEA	CFI	RMSEA	CFI	RMSEA	CFI
Australia	.061*	.915	.073	.870	.052	.876	.070	.861	.052	.934
Canada	.062*	.908	.071	.880	.051	.889	.065	.876	.053	.928
China	.041*	.918	.074*	.754	.052	.782	.056*	.841	.049	.920
Finland	.059	.910	.072	.878	.052	.884	.063	.882	.051	.938
France	.064	.880	.069	.863	.059	.825	.056	.908	.050	.925
Germany	.065	.873	.070	.878	.058	.863	.064	.873	.052*	.929
Hong Kong	.052	.910	.070	.842	.060	.761	.057	.854	.052	.929
India	.050	.917	.072	.824	.053	.817	.058	.871	.055	.916
Ireland	.061*	.914	.077	.857	.055	.873	.067	.859	.053	.935
Malaysia	.054*	.899	.073	.813	.057	.777	.069	.819	.056	.918
Mexico	.052*	.909	.068	.846	.058	.813	.058	.872	.057*	.896
Netherlands	.058*	.910	.067	.887	.055	.874	.059	.885	.051	.929
New Zealand	.060*	.914	.071	.875	.051	.886	.066	.864	.055	.918
Norway	.064*	.893	.070	.882	.054	.890	.061	.894	.056	.922
Philippines	.053*	.907	.077	.798	.056	.787	.064	.846	.059*	.909
Romania	.055*	.922	.081*	.786	.065	.786	.071	.852	.063	.906
Singapore	.057	.914	.073	.845	.051	.870	.061	.887	.053	.928
South Africa	.057*	.918	.074	.862	.052	.879	.065	.875	.053	.930
South Korea	.055	.866	.080*	.763	.048	.843	.058	.857	.053	.896
Sweden	.063	.896	.068	.890	.054	.877	.068	.875	.052	.933
UK	.064*	.909	.071	.882	.052	.890	.065	.881	.052*	.936
USA	.061	.914	.070	.881	.053	.892	.065	.873	.053	.931
Total	.059*	.914	.070	.872	.050	.890	.064	.873	.052	.932

Note. *df* = 246. The table reports fit indices of two-level hierarchical CFA models (Each FFM trait-structure is composed by its first level of 24 items and its second level of 6 facets). N = Neuroticism, E = Extraversion, O = Openness, A = Agreeableness, and C = Conscientiousness.

*Yielding an initial negative error variance in the completely free unconstrained parameter baseline model, which was amended by relaxing or constraining one parameter (changing the *df* by +-1).

Next we calculated standardized mean differences (Cohen’s *d*) between men and women for the five traits in each country (Table 2). A negative Cohen’s *d* would imply that women scored higher. The two FFM traits known to differ the most between sexes, Neuroticism and Agreeableness, showed similar patterns across 22 countries. In all countries, women provided higher scores of Neuroticism (Cohen’s *d* values ranged from -0.54 to -0.10) and Agreeableness (*d* values ranged from -0.68 to -0.22) compared to men. Likewise, similar patterns emerged in nearly all countries (with the exception of some East Asian countries) for Openness—with women again showing higher levels (Cohen’s *d* values ranged from -0.35 to -0.10). These uniform patterns in the relation between traits and the fixed variable of sex provide further support for a Similarities Hypothesis. In accordance with past research, small mean differences between men and women were observed for the traits Extraversion and Conscientiousness for all countries. The average Cohen’s *d* for Extraversion was -0.06 and 16 of the 22 countries showed women to score higher compared to men. For Conscientiousness, the average Cohen’s *d* was -0.04 and 14 of the 22 countries showed women to score higher compared to men.

**Table 2:** Descriptive statistics for standardized mean scores and sex differences for the five personality traits by country.

Country	Sample size		Means (*z*-scores)					Sex differences (*d*)				
	*N*_*Male*_	*N*_*Female*_	N	E	O	A	C	N	E	O	A	C
Australia	6557	10948	.01	.04	.06	.11	-.01	-0.40	-0.07	-0.19	-0.54	-0.08
Canada	10292	16828	.05	-.04	.02	.04	.02	-0.39	-0.07	-0.14	-0.58	-0.16
China	937	1426	-.35	-.15	-.20	-.28	.11	-0.10	0.03	-0.10	-0.32	0.01
Finland	843	949	-.04	-.37	.41	-.26	-.39	-0.39	-0.23	-0.25	-0.35	-0.07
France	649	491	-.16	.00	.48	-.17	-.03	-0.39	-0.20	-0.23	-0.54	0.01
Germany	992	938	-.24	.00	.25	-.28	.08	-0.35	-0.20	-0.16	-0.52	-0.20
Hong Kong	773	797	-.09	-.11	-.28	-.38	-.03	-0.40	0.04	0.10	-0.29	0.18
India	3129	1715	-.04	.08	.10	.08	.17	-0.30	-0.02	-0.17	-0.38	-0.01
Ireland	1281	1565	.11	.06	.15	.14	-.18	-0.41	-0.04	-0.13	-0.51	0.07
Malaysia	592	1080	.06	-.05	-.30	-.23	.00	-0.27	-0.01	-0.01	-0.22	-0.05
Mexico	637	515	-.23	.21	.13	-.27	.15	-0.45	0.04	-0.14	-0.39	0.16
Netherlands	1332	1248	-.34	.01	.26	.03	-.02	-0.41	-0.12	-0.23	-0.68	-0.15
New Zealand	1263	1750	-.04	-.10	.07	.04	-.02	-0.29	-0.20	-0.16	-0.46	-0.13
Norway	591	468	-.29	-.07	.36	.11	-.07	-0.41	-0.27	-0.28	-0.52	-0.14
Philippines	1012	1957	.10	.13	.01	-.11	-.02	-0.36	0.10	-0.04	-0.36	0.03
Romania	562	705	-.56	.17	.25	-.10	.50	-0.38	-0.02	-0.35	-0.40	0.02
Singapore	2124	2533	.07	-.01	-.30	-.19	-.16	-0.54	0.12	0.02	-0.34	0.23
South Africa	730	935	.06	.01	.08	.02	.10	-0.27	-0.04	-0.21	-0.47	-0.12
South Korea	733	816	-.10	-.22	-.30	-.47	-.13	-0.21	-0.01	-0.18	-0.36	-0.02
Sweden	1005	938	-.28	-.11	.37	.09	-.06	-0.43	-0.14	-0.27	-0.56	-0.11
UK	9388	11578	.18	-.05	.09	.01	-.23	-0.36	-0.14	-0.14	-0.61	-0.14
USA	9912	15088	-.06	.09	-.22	.03	.20	-0.42	0.01	-0.18	-0.60	-0.12
Total	55334	75268						-0.38	-0.06	-0.13	-0.53	-0.04

Note. N = Neuroticism, E = Extraversion, O = Openness, A = Agreeableness, C = Conscientiousness. A negative Cohen’s *d* implies that women scored higher. Age averages ranged from 22.7 (Singapore) to 29.7 (South Africa), with a mean of 28.0 years (*SD* 9.2).

Standardized country mean scores for the five traits were calculated and can be found in Table 2. Similar trait levels were observed for the English speaking countries, with the exception of the USA, whose Openness scores were comparatively low. Most of the countries in northern Europe followed the same general pattern, scoring comparatively high on Openness and low on Neuroticism. Romania and Finland, however, present two exceptions. In comparison to their European counterparts, Romania was markedly high in Conscientiousness and low in Neuroticism, while Finland was markedly low in both Conscientiousness and Extraversion. East Asian countries scored relatively low on all FFM traits. Although these results suggest geographical differences in personality profiles, countries were overall within less than half a standard deviation from each other. Again, we reason that this finding suggests similarities rather than differences.

**Fig 1:**
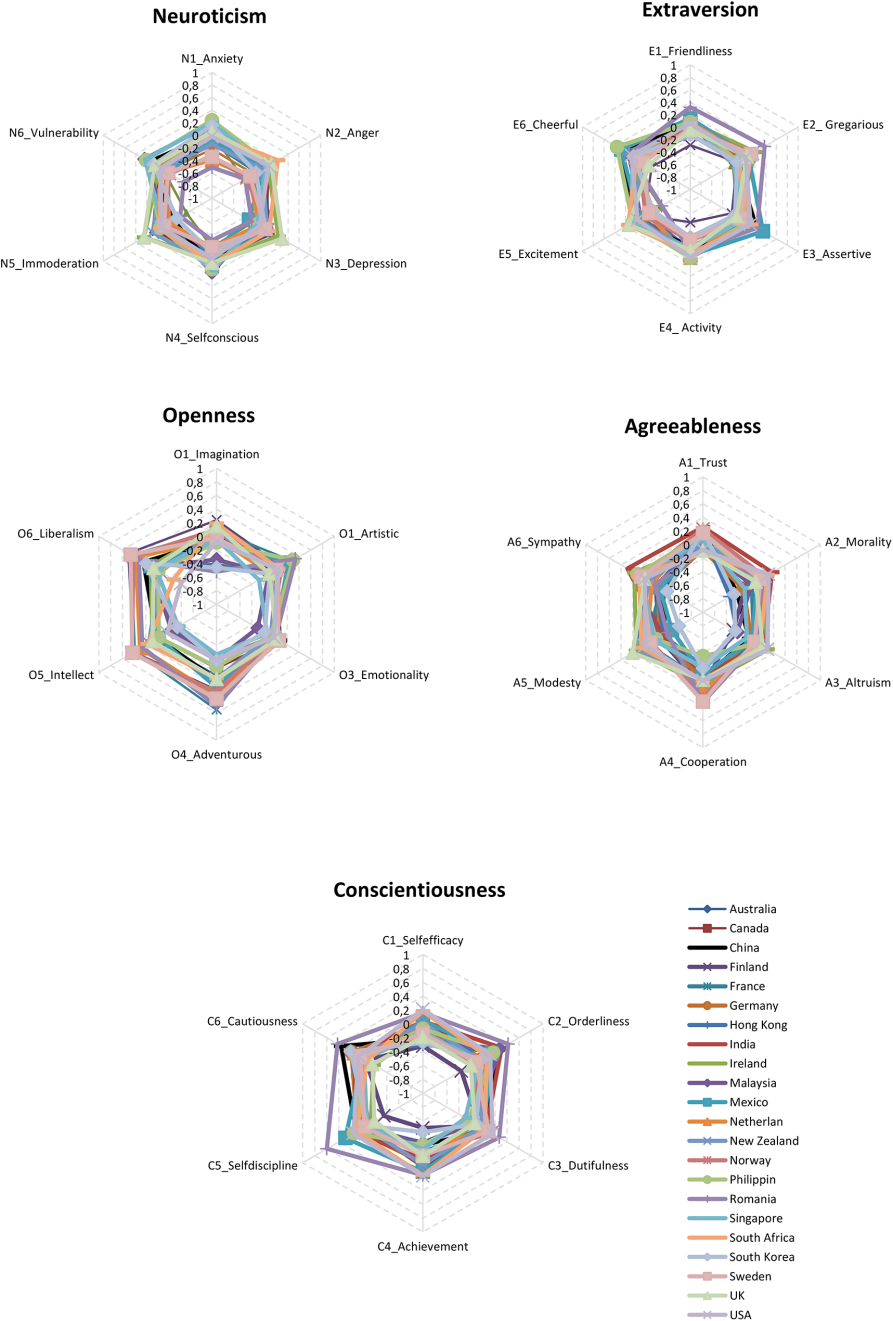
Personality trait similarities between 22 countries. The five diagrams show the standardized means (*Z*-scores) of the 5 personality structures (with each corner in the hexagons representing one of the 30 trait-facets): Neuroticism (N1-N6) top left corner, Extroversion (E1-E6) top right corner, Openness (O1-O6) middle left, Agreeableness (A1-A6) middle right, and Conscientiousness (C1-C6) at bottom.

Extending these results into 30 facet-traits, Fig 1 illustrates the similarities in the five trait profiles (five hexagons), when based on all 30 trait-facets (the five hexagons’ six corners). This portrays how countries overlap in personality profiles. Please note that the standardized facet scores constituting the hexagons are available in S2 Table.

Finally, to quantify similarities among all available personality traits between countries we conducted ANOVA-models with country as the independent variable, sex as covariate, and each one of the 30 facet-traits in turn as the dependent variable. Table 3 summarizes the F-ratio of between-country and within-country variances for each facet-trait model, as well as the explained variances (eta-squared coefficients) for country and sex, separately, for comparison. As previously noted, we considered the results as supportive of a Similarities Hypothesis if country differences accounted for less than 5% of the variance. The analyses showed that country belonging explained between 0–5% of the variation in the 30 facet-traits, with an average of 1.8%. Only one facet-trait—Liberalism—showed a substantial difference between countries (i.e., explained variance >5%). The size of country effects were overall smaller than those of sex, which ranged from 0–8%: Emotionality, sympathy, anxiety, vulnerability, altruism, and modesty (in descending order) showed effects above 5% by sex. We argue that the relatively small effects from country belonging suggest similarities, rather than differences.

**Table 3:** ANOVA-models with country effects on each of the facet-traits.

Trait	*F*(22,130601)	*Eta*^2^ (country)	*Eta*^2^ (sex)
**N1_Anxiety**	439.88	0.015	**0.055**
N2_Anger	160.08	0.013	0.012
N3_Depression	193.38	0.025	0.006
N4_Self-conscious	51.23	0.007	0.001
N5_Immoderation	224.62	0.028	0.007
N6_Vulnerability	418.22	0.013	**0.055**
E1_Friendliness	48.12	0.005	0.003
E2_ Gregarious	60.48	0.008	0.002
E3_Assertive	104.39	0.012	0.005
E4_ Activity	100.99	0.008	0.009
E5_Excitement	121.04	0.016	0.004
E6_Cheerful	146.29	0.020	0.004
O1_Imagination	117.29	0.019	0.000
O2_Artistic	170.916	0.010	0.018
**O3_Emotionality**	563.10	0.006	**0.083**
O4_Adventurous	186.89	0.027	0.003
O5_Intellect	250.47	0.027	0.013
**O6_Liberalism**	338.82	**0.053**	0.002
A1_Trust	61.23	0.010	0.000
A2_Morality	345.75	0.023	0.032
**A3_Altruism**	441.03	0.017	**0.052**
A4_Cooperation	143.76	0.011	0.013
**A5_Modesty**	495.47	0.026	**0.051**
**A6_Sympathy**	449.88	0.015	**0.058**
C1_Self-efficacy	145.32	0.024	0.000
C2_Orderliness	139.52	0.021	0.002
C3_Dutifulness	150.31	0.015	0.010
C4_Achievement	208.95	0.022	0.011
C5_Self-discipline	121.68	0.019	0.001
C6_Cautiousness	97.45	0.013	0.003

Note. The *Eta*^*2*^ columns show the percentage of explained variance by the main effect of country and the covariate sex in the same model, for each one of the facet-traits. Country explained on average 1.8%.

Bold letters indicate strong effects, beyond the Similarities Hypothesis (>5%).

## Discussion

The current study used a 120-item FFM personality scale with some of the largest country samples to date. We approached the data with a proposed Similarities Hypothesis, suggesting that similarities rather than differences in personality traits would be most characteristic of the results. The results showed similar model fits for each of the five trait factor-structures across countries, as well as uniform patterns in trait relationships with sex. Furthermore, the aggregate personality levels of most countries were within half a standard deviation of each other (Fig 1). More detailed analyses showed that country belonging explained on average 1.8% of variance in personality traits (Table 3). Using a cut-off of 5% explained variance by country [26], the present results are in accordance with our proposed Similarities Hypothesis.

### A personality similarities hypothesis

The current findings add to the mounting evidence demonstrating small country differences in aggregate personality traits [4, 23]. In the current study, on average 1.8% of the variance in personality traits could be accounted for by country belonging. Put differently, within-country differences in personality traits are of more interest than between-country differences. This corresponds with previous research showing that within country differences in personality are up to three times larger than between country differences [21, 24]. Additionally, cross country personality studies often lack solid measurement invariance [32, 33]. A lack of measurement invariance can imply that the already low explained variance per facet-trait may be smaller still if more valid between-country measures of personality were used [33]. While we acknowledge genuine country differences in country aggregate personality traits, these are likely to be small. We therefore suggest that it may be time to consider country aggregate personalities in terms of similarities, rather than differences.

A personality Similarities Hypothesis could also account for the inconsistent results between studies in terms of country rank ordering of personality traits. Although many patterns were replicated in the current study—for instance, English speaking countries and European countries were higher than East Asian countries in terms of Openness—many other patterns were not. For example, against stereotypic beliefs, McCrae [30] found Scandinavian countries to score highest on Extraversion. In the current study, however, Scandinavian countries were in the lower half of countries ranked by Extraversion. As another example, China and France have previously shown above average scores on Neuroticism [23], but in the current study they were below average. A Similarities Hypothesis would suggest that in the absence of large country differences, it is unlikely that consistent patterns of country rank orders will emerge between studies.

### Limitations

One concern about internet surveys is the lack of control and knowledge of who the respondents are. In the present data collection, participants had to actively find the internet site and complete the questionnaires with feedback as their only incentive. This means that despite the generous number of respondents from each country these may not be representative samples, due to self-selection biases. For the purpose of comparing countries, the similar age of respondents, as well as the mostly similar proportions of females, speaks in support of the validity of the study. Furthermore, it is notable that that we were able to affirm a Similarities Hypothesis, despite the presumably heterogenic country samples, where differences may have been driven by other factors than country-belonging.

Another major concern with the internet survey was that only English-speaking participants were able to participate. First, this may imply higher socio-economic status and younger age, especially in non-English speaking countries, thus explaining country-characteristic trait levels. Second, this could also lower validity since English is not the native language for many country samples. However, it seems unlikely that people with poor English-skills would actively seek out an extensive internet survey in English. Furthermore, the IPIP-NEO is specifically designed to be reader friendly as it is composed of short, easy to understand, concrete items (e.g.“…yell at others, “…seek adventure”, and “…tell the truth”).

It may instead be that our country samples were unrepresentatively homogenous, which may even be viewed as a strength when attempting to elicit differences based on country belonging. A reasonable estimation is that the trait Openness, which correlates broadly knowledge and acquisition of languages, was overrepresented, since this is known to characterize people interested in psychology [34].

### Conclusion

It has been suggested that country aggregate personality traits can provide an insight into the culture of a country [23]. This idea fits well with the commonly held beliefs about strong and reliable differences in the national characteristics of countries [35]. The results of the present study challenge these intuitive assumptions. Until more convincing evidence is found, the most parsimonious interpretation for the current findings, and for much of past research, may be a cross-country personality Similarities Hypothesis.

## Supporting information

S1 Table Descriptive statistics (means, standard deviations, and alpha consistencies) for the five trait factors across countries. (DOCX)

S2 Table Standardized z-scores for the 30 facet traits by country. (DOCX)
