# Supplementary material for: Personality traits across countries: Support for similarities rather than differences
Source: PLoS One. 2017 Jun 16;12(6):e0179646. doi: 10.1371/journal.pone.0179646 (PMC5473578; doi:10.1371/journal.pone.0179646)
Supplement: S1 Table — (DOCX) [file pone.0179646.s001.docx]

**S1 Table. Descriptive statistics (means, standard deviations, and alpha consistencies) for the five trait factors across countries.**

| Country |  | N |  |  | E |  |  | O |  |  | A |  |  | C |  |  |
| --- | --- | --- | --- | --- | --- | --- | --- | --- | --- | --- | --- | --- | --- | --- | --- | --- |
|  | N | M | SD | α | M | SD | α | M | SD | α | M | SD | α | M | SD | α |
| Australia | 17505 | 11.31 | 2.72 | 0.91 | 13.59 | 2.36 | 0.89 | 14.26 | 1.92 | 0.81 | 15.03 | 1.99 | 0.85 | 14.43 | 2.39 | 0.90 |
| Canada | 27120 | 11.41 | 2.67 | 0.90 | 13.40 | 2.38 | 0.89 | 14.16 | 1.97 | 0.81 | 14.89 | 2.07 | 0.86 | 14.51 | 2.36 | 0.90 |
| China | 2363 | 10.36 | 2.03 | 0.84 | 13.13 | 1.81 | 0.82 | 13.75 | 1.59 | 0.73 | 14.25 | 1.61 | 0.77 | 14.73 | 2.09 | 0.88 |
| Finland | 1792 | 11.16 | 2.53 | 0.90 | 12.62 | 2.49 | 0.90 | 14.94 | 1.86 | 0.81 | 14.29 | 1.92 | 0.84 | 13.54 | 2.33 | 0.89 |
| France | 1140 | 10.86 | 2.45 | 0.88 | 13.49 | 2.32 | 0.88 | 15.07 | 1.77 | 0.77 | 14.47 | 2.09 | 0.86 | 14.40 | 2.20 | 0.87 |
| Germany | 1930 | 10.64 | 2.42 | 0.89 | 13.49 | 2.36 | 0.89 | 14.62 | 1.95 | 0.82 | 14.25 | 1.93 | 0.84 | 14.65 | 2.27 | 0.89 |
| Hong Kong | 1570 | 11.05 | 2.29 | 0.89 | 13.22 | 1.99 | 0.86 | 13.59 | 1.59 | 0.74 | 14.05 | 1.67 | 0.81 | 14.38 | 2.22 | 0.90 |
| India | 4844 | 11.18 | 2.43 | 0.87 | 13.68 | 2.11 | 0.85 | 14.33 | 1.73 | 0.75 | 14.97 | 1.85 | 0.82 | 14.86 | 2.40 | 0.90 |
| Ireland | 2846 | 11.56 | 2.84 | 0.91 | 13.62 | 2.36 | 0.88 | 14.43 | 1.97 | 0.81 | 15.09 | 2.03 | 0.85 | 14.03 | 2.60 | 0.91 |
| Malaysia | 1672 | 11.43 | 2.28 | 0.88 | 13.37 | 2.01 | 0.85 | 13.56 | 1.61 | 0.72 | 14.34 | 1.79 | 0.82 | 14.46 | 2.36 | 0.91 |
| Mexico | 1152 | 10.68 | 2.41 | 0.87 | 13.98 | 2.09 | 0.85 | 14.40 | 1.84 | 0.85 | 14.26 | 1.98 | 0.83 | 14.82 | 2.29 | 0.88 |
| Netherlands | 2580 | 10.38 | 2.38 | 0.89 | 13.50 | 2.32 | 0.90 | 14.65 | 1.86 | 0.82 | 14.87 | 1.82 | 0.84 | 14.41 | 2.15 | 0.88 |
| New Zealand | 3013 | 11.19 | 2.60 | 0.90 | 13.25 | 2.34 | 0.89 | 14.28 | 1.90 | 0.81 | 14.90 | 1.98 | 0.85 | 14.41 | 2.34 | 0.90 |
| Norway | 1059 | 10.51 | 2.47 | 0.89 | 13.33 | 2.46 | 0.90 | 14.84 | 1.99 | 0.83 | 15.03 | 1.96 | 0.85 | 14.29 | 2.31 | 0.89 |
| Philippines | 2969 | 11.54 | 2.32 | 0.88 | 13.78 | 1.93 | 0.84 | 14.16 | 1.55 | 0.73 | 14.58 | 1.85 | 0.83 | 14.41 | 2.39 | 0.91 |
| Romania | 1267 | 9.80 | 2.59 | 0.91 | 13.89 | 1.95 | 0.84 | 14.62 | 1.68 | 0.77 | 14.60 | 1.94 | 0.85 | 15.66 | 2.37 | 0.92 |
| Singapore | 4657 | 11.47 | 2.33 | 0.89 | 13.47 | 2.03 | 0.87 | 13.56 | 1.75 | 0.80 | 14.42 | 1.89 | 0.85 | 14.08 | 2.17 | 0.89 |
| South Africa | 1665 | 11.43 | 2.72 | 0.90 | 13.51 | 2.36 | 0.88 | 14.30 | 1.95 | 0.81 | 14.86 | 2.09 | 0.86 | 14.71 | 2.36 | 0.89 |
| South Korea | 1549 | 11.00 | 2.03 | 0.85 | 12.97 | 1.91 | 0.85 | 13.56 | 1.71 | 0.77 | 13.87 | 1.70 | 0.80 | 14.15 | 1.95 | 0.86 |
| Sweden | 1943 | 10.53 | 2.49 | 0.89 | 13.23 | 2.51 | 0.90 | 14.86 | 1.91 | 0.81 | 14.99 | 2.12 | 0.88 | 14.32 | 2.30 | 0.89 |
| UK | 20966 | 11.76 | 2.72 | 0.90 | 13.36 | 2.43 | 0.90 | 14.30 | 1.97 | 0.82 | 14.83 | 2.09 | 0.86 | 13.91 | 2.43 | 0.90 |
| USA | 25000 | 11.11 | 2.67 | 0.90 | 13.70 | 2.37 | 0.89 | 13.71 | 2.05 | 0.82 | 14.87 | 2.02 | 0.85 | 14.94 | 2.34 | 0.90 |
| Total | 130602 | 11.27 | 2.64 | 0.90 | 13.49 | 2.34 | 0.89 | 14.13 | 1.96 | 0.81 | 14.80 | 2.02 | 0.85 | 14.46 | 2.38 | 0.90 |

Note. N = Neuroticism, E = Extraversion, O = Openness, A = Agreeableness, and C = Conscientiousness.
