# Supplementary material for: Personality traits across countries: Support for similarities rather than differences
Source: PLoS One. 2017 Jun 16;12(6):e0179646. doi: 10.1371/journal.pone.0179646 (PMC5473578; doi:10.1371/journal.pone.0179646)
Supplement: S2 Table — (DOCX) [file pone.0179646.s002.docx]

**S2 Table. Standardized z-scores for the 30 facet traits by country.**

|  | N1_Anxiety | N2_Anger | N3_Depression | N4_Selfconscious | N5_Immoderation | N6_Vulnerability | E1_Friendliness | E2_ Gregarious | E3_Assertive | E4_ Activity | E5_Excitement | E6_Cheerful | O1_Imagination | O2_Artistic | O3_Emotionality | O4_Adventurous | O5_Intellect | O6_Liberalism | A1_Trust | A2_Morality | A3_Altruism | A4_Cooperation | A5_Modesty | A6_Sympathy | C1_Selfefficacy | C2_Orderliness | C3_Dutifulness | C4_Achievement | C5_Selfdiscipline | C6_Cautiousness |
| --- | --- | --- | --- | --- | --- | --- | --- | --- | --- | --- | --- | --- | --- | --- | --- | --- | --- | --- | --- | --- | --- | --- | --- | --- | --- | --- | --- | --- | --- | --- |
| Australia | -.06 | .00 | .04 | -.01 | .09 | -.01 | -.02 | .03 | -.01 | .07 | .08 | .02 | .05 | .02 | .05 | .05 | .06 | -.01 | .03 | .09 | .08 | .05 | .09 | .08 | .03 | -.03 | .00 | .05 | -.03 | -.05 |
| Canada | .06 | .05 | -.01 | .05 | .03 | .02 | -.03 | -.02 | .00 | -.04 | -.03 | -.02 | .04 | .01 | .04 | -.06 | -.01 | .05 | -.02 | .08 | .05 | .00 | .07 | .00 | .07 | -.02 | .05 | .07 | -.02 | -.03 |
| China | .03 | -.41 | -.24 | -.22 | -.53 | -.11 | .03 | -.21 | -.12 | -.06 | -.45 | .20 | -.41 | .02 | -.07 | -.19 | -.30 | .27 | .20 | -.42 | -.21 | -.03 | -.35 | -.31 | -.22 | .24 | .05 | -.25 | .11 | .36 |
| Finland | -.06 | -.13 | .13 | -.04 | -.07 | -.01 | -.29 | -.13 | -.23 | -.47 | -.16 | -.28 | .24 | .11 | -.04 | .26 | .41 | .50 | .01 | -.28 | -.48 | .11 | -.25 | -.19 | -.32 | -.36 | -.06 | -.50 | -.35 | -.05 |
| France | -.13 | -.18 | .01 | -.02 | -.17 | -.18 | -.02 | -.04 | .06 | .02 | .01 | -.03 | .09 | .28 | -.07 | .55 | .39 | .45 | -.07 | -.17 | -.13 | .02 | -.05 | -.28 | -.12 | -.05 | .00 | -.15 | .00 | .15 |
| Germany | -.22 | -.23 | -.13 | -.17 | -.13 | -.14 | -.05 | -.01 | .17 | .11 | -.18 | -.02 | -.10 | .02 | -.04 | .34 | .27 | .40 | -.08 | -.31 | -.17 | -.02 | -.24 | -.25 | -.05 | .00 | .06 | .00 | .09 | .19 |
| Hong Kong | .12 | -.18 | -.11 | -.05 | -.26 | .11 | .01 | -.08 | -.20 | -.03 | -.20 | .04 | -.41 | -.13 | -.17 | -.22 | -.27 | .21 | .19 | -.54 | -.42 | -.20 | -.32 | -.22 | -.30 | .20 | -.16 | -.29 | .00 | .19 |
| India | .02 | .06 | -.19 | .08 | -.29 | .15 | .05 | -.14 | .22 | -.08 | .04 | .28 | -.06 | .00 | .07 | .11 | .01 | .24 | .26 | .18 | .03 | -.10 | -.31 | .29 | .04 | .31 | .05 | -.05 | .13 | .13 |
| Ireland | .01 | .03 | .22 | -.02 | .25 | -.03 | .08 | .20 | -.09 | -.05 | .14 | -.08 | .13 | .04 | .02 | .15 | .09 | .12 | .02 | .02 | .10 | .08 | .14 | .17 | -.13 | -.11 | -.11 | -.09 | -.13 | -.18 |
| Malaysia | .19 | .02 | -.20 | .18 | -.18 | .24 | .03 | -.15 | -.13 | .00 | -.08 | .14 | -.34 | -.11 | -.30 | -.18 | -.23 | .10 | -.01 | -.18 | -.41 | -.21 | -.22 | .10 | -.23 | .28 | -.14 | -.28 | .00 | .15 |
| Mexico | -.05 | -.05 | -.32 | -.14 | -.21 | -.19 | .14 | .08 | .35 | .06 | -.05 | .28 | -.06 | .22 | -.02 | .22 | -.01 | .12 | .02 | -.07 | -.17 | -.08 | -.47 | -.26 | .05 | .15 | .01 | .13 | .29 | .00 |
| Netherland | -.39 | -.31 | -.14 | -.21 | -.21 | -.18 | .08 | .06 | -.05 | .10 | -.20 | .02 | -.02 | -.09 | .07 | .34 | .30 | .37 | .20 | -.08 | -.05 | .18 | -.03 | -.13 | -.18 | .00 | .01 | -.12 | -.03 | .15 |
| New Zealand | -.14 | -.08 | .02 | .07 | .09 | -.09 | -.12 | -.10 | -.11 | -.04 | .01 | -.07 | .07 | .04 | -.08 | .10 | .15 | -.04 | .03 | .07 | -.03 | .06 | .09 | -.07 | .01 | -.05 | -.02 | .03 | -.03 | -.01 |
| Norway | -.33 | -.36 | .02 | -.18 | -.15 | -.22 | -.01 | .15 | -.07 | -.17 | -.17 | -.06 | .10 | .07 | -.02 | .29 | .44 | .41 | .23 | .05 | -.11 | .27 | -.11 | .05 | -.07 | -.20 | .14 | -.14 | -.10 | .10 |
| Philippines | .24 | .07 | -.17 | .13 | -.05 | .21 | .08 | -.11 | .05 | .10 | .07 | .36 | -.09 | .30 | -.08 | -.08 | -.02 | -.01 | .16 | -.06 | -.08 | -.35 | -.20 | .12 | -.07 | .17 | -.24 | -.25 | .15 | .02 |
| Romania | -.51 | -.39 | -.27 | -.33 | -.42 | -.48 | .32 | .38 | .24 | .01 | -.47 | .13 | -.52 | .34 | .00 | .50 | .26 | .32 | -.08 | .00 | -.08 | .33 | -.39 | -.18 | .16 | .42 | .27 | .18 | .60 | .42 |
| Singapore | .20 | .00 | -.17 | .15 | -.10 | .23 | .07 | -.19 | -.02 | -.04 | .10 | .07 | -.04 | -.28 | -.14 | -.26 | -.35 | .02 | .08 | -.29 | -.25 | -.18 | -.17 | .05 | -.12 | -.06 | -.29 | -.19 | -.07 | -.02 |
| South Africa | .01 | .21 | .05 | .05 | -.05 | -.05 | -.12 | -.12 | .13 | .03 | .15 | .00 | .19 | .05 | .05 | .09 | .17 | -.27 | -.13 | .10 | -.03 | .02 | .05 | .09 | .18 | .07 | .03 | .18 | .10 | -.07 |
| South Korea | .16 | -.19 | -.02 | -.13 | -.35 | .07 | -.12 | -.13 | -.17 | -.23 | -.31 | .05 | -.46 | -.10 | -.18 | -.17 | -.33 | .20 | .21 | -.47 | -.45 | -.17 | -.59 | -.39 | -.27 | -.02 | -.11 | -.44 | -.07 | .20 |
| Sweden | -.34 | -.30 | -.02 | -.22 | -.12 | -.19 | -.02 | .13 | -.07 | -.19 | -.24 | -.13 | -.02 | .01 | .06 | .39 | .43 | .46 | .17 | -.02 | -.12 | .32 | -.10 | .05 | -.14 | -.11 | .09 | -.10 | -.03 | .04 |
| UK | .06 | .09 | .29 | .03 | .25 | .05 | -.04 | .05 | -.14 | -.02 | .14 | -.23 | .15 | -.10 | .02 | .10 | .09 | .07 | -.09 | -.10 | .02 | .00 | .20 | -.01 | -.19 | -.20 | -.14 | -.10 | -.19 | -.16 |
| USA | -.02 | .04 | -.12 | -.04 | -.07 | -.06 | .06 | .03 | .12 | .09 | -.02 | .09 | -.08 | .04 | -.02 | -.18 | -.16 | -.39 | -.08 | .12 | .10 | .01 | -.01 | -.01 | .21 | .12 | .16 | .19 | .16 | .07 |
